# A tissue factor-cascade-targeted strategy to tumor vasculature: a combination of EGFP-EGF1 conjugation nanoparticles with photodynamic therapy

**DOI:** 10.18632/oncotarget.12922

**Published:** 2016-10-26

**Authors:** Wei Shi, Yanxue Yin, Yao Wang, Bo Zhang, Pei Tan, Ting Jiang, Heng Mei, Jun Deng, Huafang Wang, Tao Guo, Zhiqing Pang, Yu Hu

**Affiliations:** ^1^ Institute of Hematology, Union Hospital, Tongji Medical College, Huazhong University of Science & Technology, Wuhan, Hubei, China; ^2^ Targeted Biotherapy Key Laboratory of Ministry of Education, Wuhan, Hubei, China; ^3^ Department of Pediatrics, Tongji Hospital, Tongji Medical College, Huazhong University of Science & Technology, Wuhan, Hubei, China; ^4^ Department of Pharmaceutics, School of Pharmacy, Fudan University, Shanghai, China

**Keywords:** EGFP-EGF1, tissue factor, TF-cascade-targeted drug delivery system, photodynamic therapy

## Abstract

Tumor requires tumor vasculature to supply oxygen and nutrients so as to support its continued growth, as well as provide a main route for metastatic spread. In this study, a TF-cascade-targeted strategy aiming to disrupt tumor blood vessels was developed by combination of TF-targeted HMME-loaded drug delivery system and PDT. PDT is a promising new modality in the treatment of cancers, which employs the interaction between a tumor-localizing photosensitizer and light of an appropriate wavelength to bring about ROS-induced cell death. *In vitro* results showed that protein EGFP-EGF1modification could significantly contribute to the uptake of nanoparticles by TF over-expressed BCECs. *In vivo* multispectral fluorescent imaging, the EGFP-EGF1 conjugated nanoparticles showed significantly higher accumulation in tumor tissues than non-conjugated ones. Tumor tissue slides further presented that EGFP-EGF1 conjugated nanoparticles showed significantly higher accumulation in tumor vasculature than non-conjugated ones. *In vitro* study demonstrated that PDT increased TF expression of BCECs. *In vivo* imaging, ex vivo imaging and tumor tissue slides showed that PDT further contribute EGFP-EGF1-NP accumulation in tumor. These promising results indicated that PDT enhanced EGFP-EGF1modified PEG-PLGA nanoparticle accumulation in tumor vaculature. Considering that EGFP-EGF1 conjugation enhanced nanoparticles uptake by TF over-expressed endothelium and PDT increased endothelium TF expression. We conclude that PDT triggered a TF cascade targeted effect. A combination of both EGFP-EGF1 modification and PDT provided a positive feed-back target effect to tumor vessels and might have a great potential for tumor therapy.

## INTRODUCTION

Tumor angiogenesis and vasculature is the pathological basis of their proliferation and metastasis. Targeting vascular network is therefore an attractive approach for the treatment of human malignancies [1, 2]. Concentration of therapeutic vascular targeting has been adopted in anti-angiogenic approaches so far, which prevents the neovascularization but don't damage well-established vessels. Hence selective shutdown of the established tumor vasculature, maybe a promising strategy. There are two ways of anti-vasculature therapies up to date. First, Antibodies, peptides or grow factors were conjugated with drugs, toxins, photosensitizer, cytokines or tissue factor, or incorporated into vectors for gene delivery [3–7]. Second, small molecule vasculature disrupting agents (VDAs) selectively disrupt tumor vessels, including combretastatins and drugs related to 5,6-dimethylxanthenone-4-acetic acid (DMXAA) which are the two main groups of low-weight molecule anti-vasculature drugs applied in preclinical and clinical trials [8].

Tissue factor (TF) is aberrantly expressed on the surface of the tumor vasculature rather than normal vasculature. TF could served as a potential targeted molecular for targeting tumor vasculature. In our previous study, we have successfully established a delivery to TF over-expressed cells. EGFP-EGF1 fusion protein, derived from FVII, which contained special FVII binding domain of TF without procoagulant activity, was conjugated to PEG-PLA nanoparticles. It has been proven to have efficient target effect to neovascular and tumor cells of brain glioma which over expressed TF. However, target therapy based on antibodies, peptides, grow factors or our EGFP-EGF1 protein conjugation may miss the precise location. So target therapy involved PDT which gives “a second location” appeared with more accurate target efficacy. It only effects by strictly tumor-focused exposure to laser light, which may be highly specific because of largely improving tumor imaging modalities [17, 18]. In human lung cancer and breast cancer, especially when breast cancer became chemoresistant, FVII bounding photosensitizers showed efficient and safe [7, 15, 16]. But challenges remained. The killing mechanisms depend on that ROS directly induce cell death. However, it requires hours between administration of photosensitizer and radiation, called drug-light interval (DLI), to make drug distribute in parenchyma cells. The long interval increases risk of light toxicity. Anti-vasculature PDT which aims to target tumour vessels rather than its parenchyma may served as a alternative therapeutic approach.

We established a TF-cascade-targeted strategy to target tumor vasculature. It was supposed to take advantage of combination of TF-targeted nanoparticles and PDT to cascade recruit TF-targeted nanoparticles to tumor vasculature, eventually disrupting tumor vasculature. Not only TF-cascade-targeted efficacy was made in the strategy, but also TF-targeted nanoparticles would precisely direct the tumor vasculature because of PDT. TF-targeted nanoparticles can target tumor vaculature like most target nanoparticles in current study but the difference is they are almost “silent” after administration. And it was relied on PDT toactivate. PDT can be accurately conducted on wanted site to activate the nanoparticles. PDT also provide safety to normal organs because the nanoparticles in the part without PDT would be inactivate.

Here we established a TF-cascade-targeted drug delivery system——EGFP-EGF1-NP loaded HMME (ENP-HMME) for tumor therapy. EGFP-EGF1 served as the targeting molecular. We hypothesized that ROS was intentionally produced during PDT and caused vascular endothelial injury, inducing TF expression on the endothelium of tumor vasculature. Then ENP-HMME gathered more in tumor blood vessels over-expressing TF, because of the ability of targeting TF. During the process of PDT, TF further released, which recruit more TF-targeted ENP-HMME. Targeting property of fluorescence-labeled ENP and the forming ability of TF by BCECs were investigated both *in vitro* and *in vivo* and compared with those of unmodified NP-HMME.

## RESULTS

### Characterization of nanoparticles

**Figure 1:**
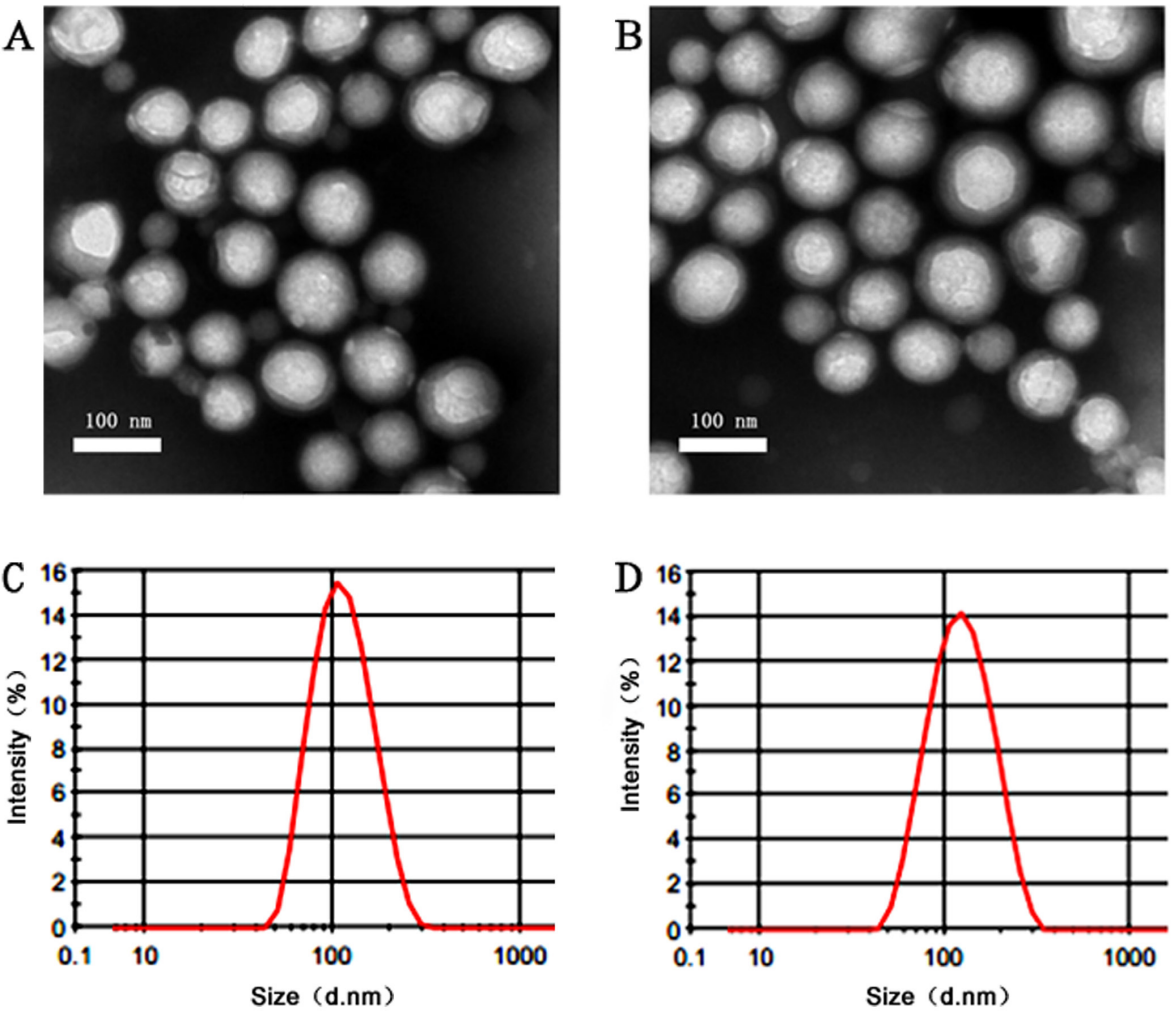
Characterastics of different nanoparticles The observation under TEM of NP **A**. and EGFP-EGF1-NP**B**.; Size distribution of NP **C**. and EGFP-EGF1-NP **D**. by dynamic light scattering(DLS) with He-Ne laser at 632.8 nm.

As depicted in Figure 1, TEM observation showed that the NP-HMME AND ENP-HMME were consistent with the size and of uniform shape. NPs and ENPs loaded HMME which were labeled or unlabeled with coumarin-6 or Dir are all between 100nm and 120nm with a narrow size distribution (polydispersity index, PdI < 0.2). The HMME loaded NPs had an average diameter of about 103.2 nm and the diameter was increased to approximately 116.4 nm after EGFP-EGF1 conjugation. After coumarin-6 or Dir encapsulation, EGFP-EGF1-NP had an average diameter of 117.9 nm and 118.2nm, respectively. There was no significant difference in particle size between EGFP-EGF1-conjugated nanoparticles and non-conjugated ones and between loaded coumarin-6/Dir and its non-loaded counterpart. Concentrations of HMME (wave length 395nm), coumarin-6 or Dir (wave length 750nm) in nanoparticles were determined based on their respective absorbance spectra in acetonitrile. No differences were observed in drug entrapment efficiency between EGFP-EGF1 conjugated nanoparticles and non-conjugated counterparts (HMME 4.24 ±0.016 *versus* 3.75 ± 0.021, Dir 1.63 ± 0.008 *versus* 1.66 ± 0.005, Coumarin-6 0.270 ± 0.011 *versus* 0.275 ± 0.003, mg/g).

### Uptake characteristic of ENPs by TF over-expressed BCECs

**Figure 2:**
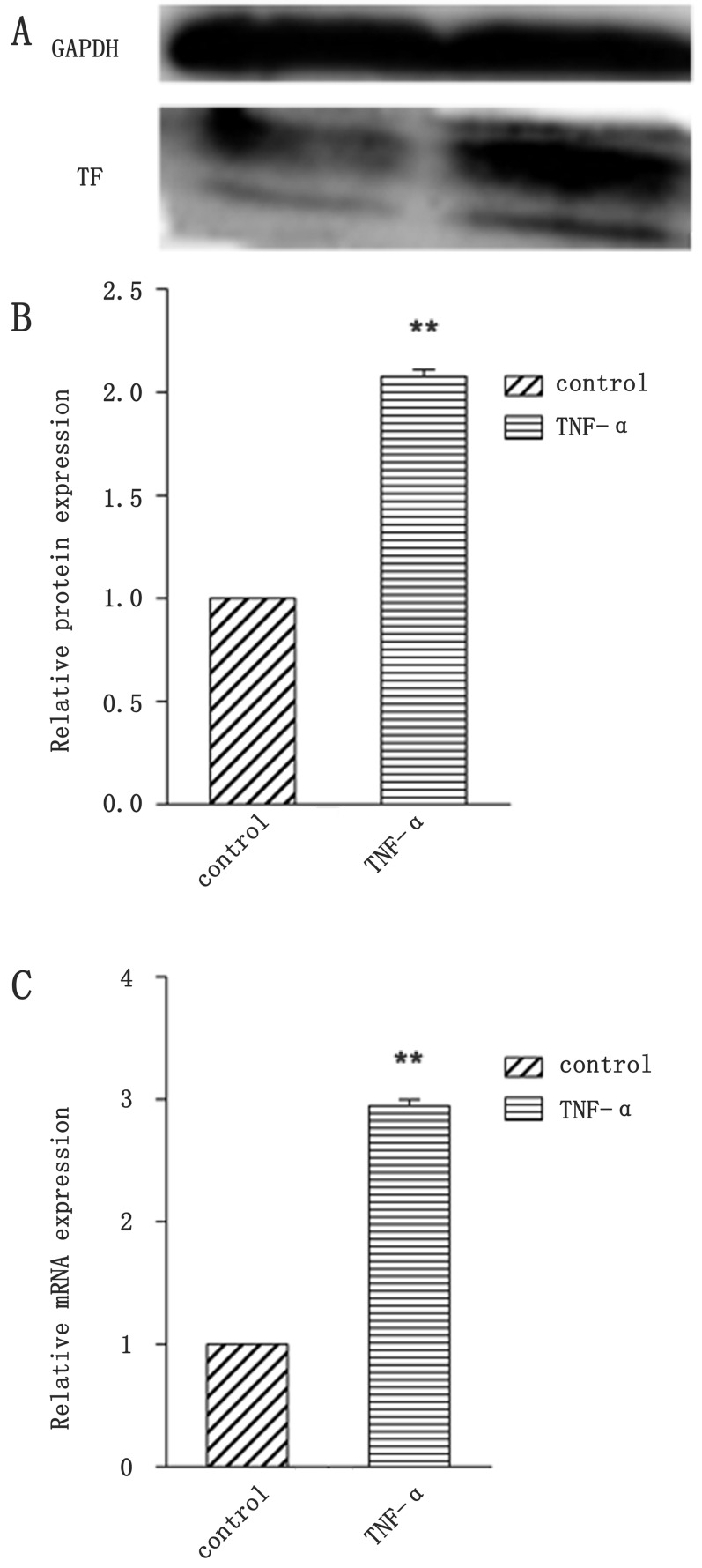
Identification of BCECs TF expression by TNF-α stimulation **A**. TF protein expression of BCECs was analyzed by western blotting; **B**. Analysis of gray of picture A and normalized by GAPDH and blank BCECs; **C**. Relative fold of TF mRNA after normalizing to GAPDH mRNA and blank BCECs. Data are expressed as mean ± SEM (*n* = 3); **p < 0.01, compared with control group.

**Figure 3:**
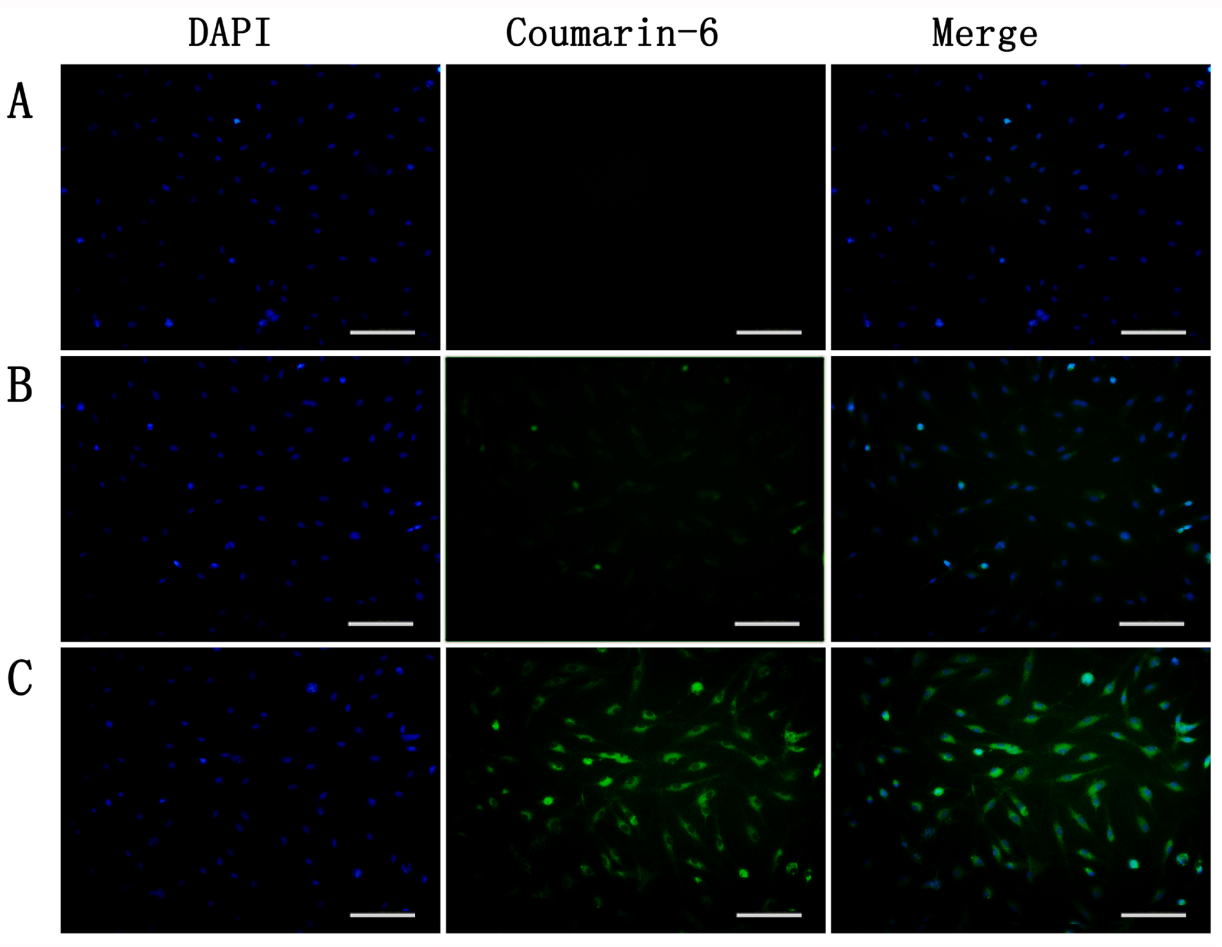
Uptake of coumarin-6-labeled nanoparticles by TNF-α-stimulated BCECs at 37 o**C** for 3h was observed under microscopy. **A**. blank, **B**. NP and **C**. EGFP-EGF1-NP. Bar = 100um.

**Figure 4:**
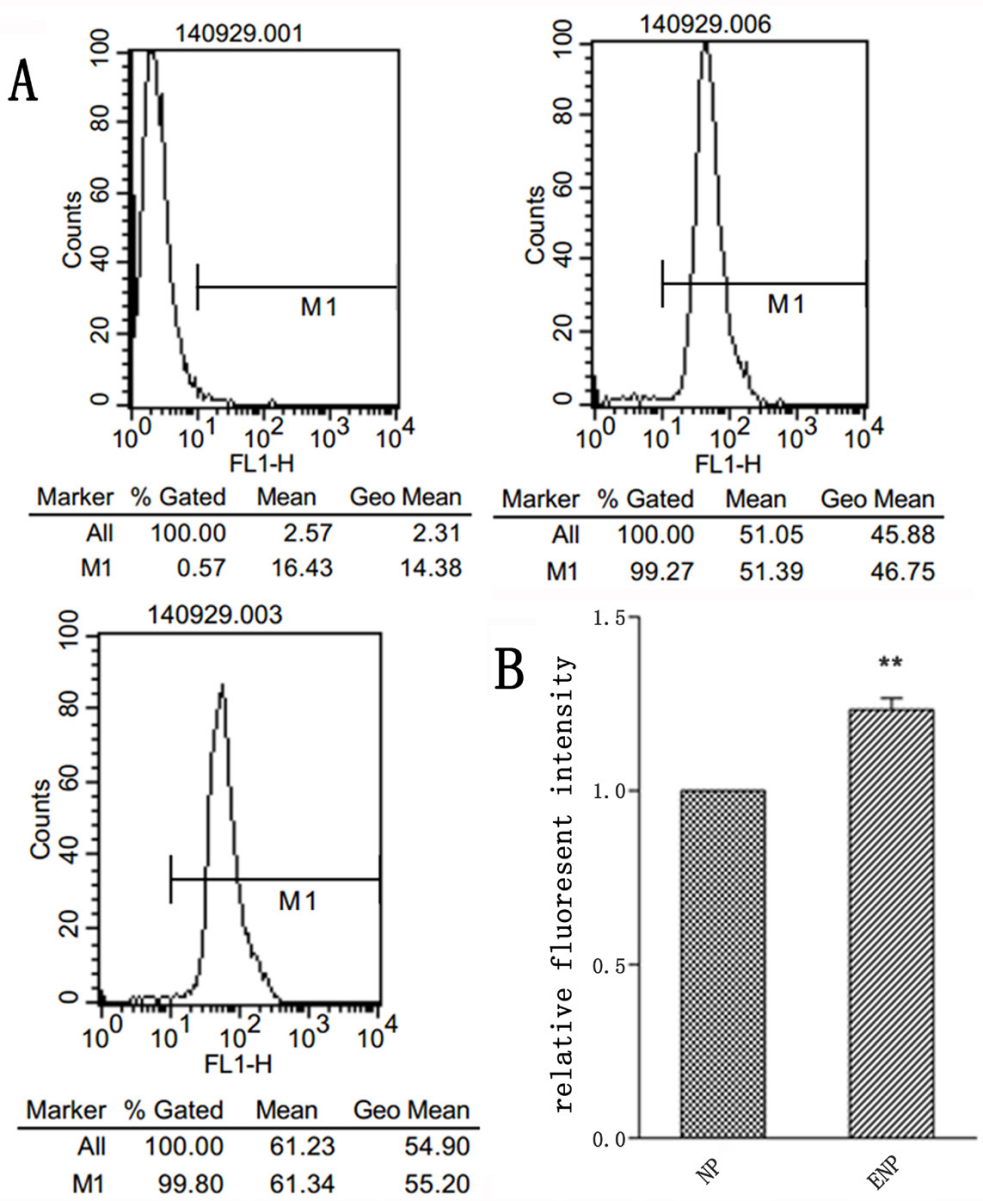
Uptake of coumarin-6-labeled nanoparticles by TNF-α-stimulated BCECs at 37 o**C** for 3h was investigated by flowcytometry. **A**. was tested by flowcytometry; **B**. was analysis of flowcytometry data. Data are expressed as mean ± SEM (*n* = 3); **p < 0.01, compared with control group.

To investigate the uptake of nanoparticles, BCECs co-incubated with TNF-α to induce TF expression. Figure 2 showed that TF expression of BCECs raises to more than 2 folds of non-stimulated ones. Coumarin-6 (green) was encapsuled as a fluorescence indicator to track nanoparticles. TNF-α stimulated BCECs incubated with coumarin-6-labeled ENP revealed significantly higher fluorescence intensity than that incubated with coumarin-6-labeled NP after incubation for 3h (Figure 3B, 3C). An intense green fluorescent signal was found both in cytoplasm and nucleus. However nanoparticles entered cytoplasm more. Flowcytometry also demonstrated uptake of ENP was more prominent, consistent with the qualitative analyses (Figure 4). These data indicated that protein EGFP-EGF1 conjugation to the nanoparticles could significantly contribute to the uptake of nanoparticles by BCECs over-expressing TF, as EGFP-EGF1 on the surface of nanoparticles could specifically recognize TF.

### *In-vitro* TF expression post-PDT

**Figure 5:**
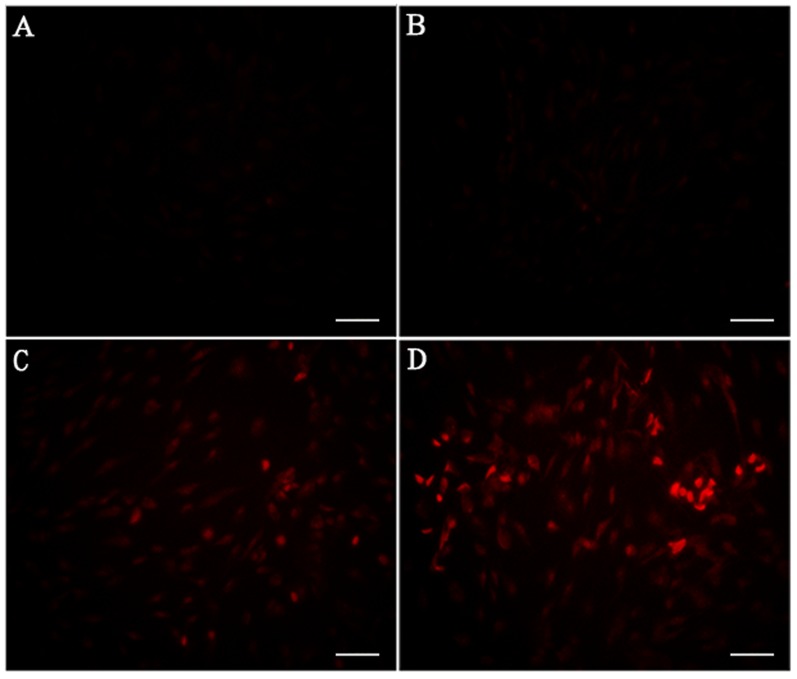
Estimation of ROS production in BCECs post-PDT **A**. blank; **B**. single radiation; **C**. NP plus PDT; **D**. EGFP-EGF1-NP plus PDT. Bar = 100um.

**Figure 6:**
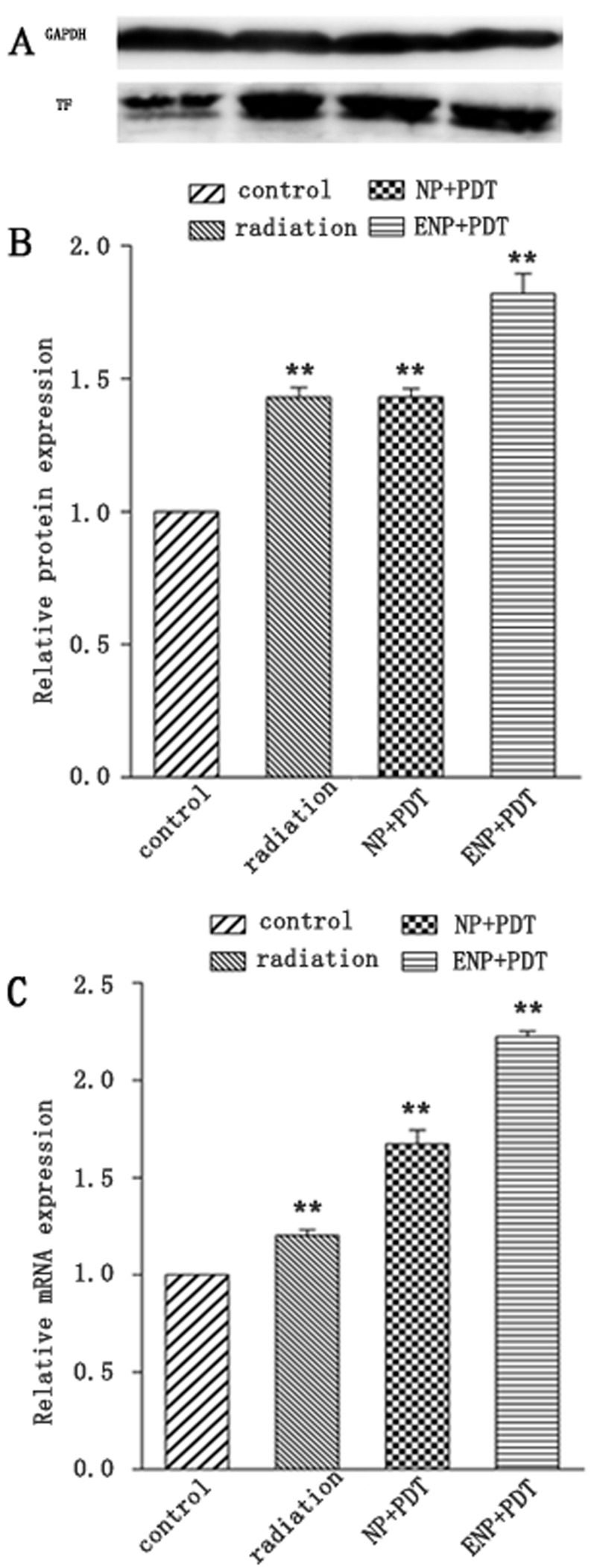
TF expression of BCECs post-PDT **A**.TF protein expression was analysized by western blotting and normalized by GAPDH and blank BCECs; **B**. analysis of gray of picture A; **C**. relative fold of TF mRNA after normalizing to GAPDH mRNA and blank BCECs; Data are expressed as mean ± SEM (*n* = 3); **p < 0.01, compared with control group.

The TF expression of BCECs post-PDT with various HMME formulations were also investigated both at the level of transcription and post-transcription. At 2-hour post-PDT, we observed that both single radiation and PDT increased TF expression at transcription level in BCECs (Figure 6C). At the same time, ENP-HMME showed stronger enhancement of inducing TF expression than NP-HMME post-PDT (Figure 6). Similarly, ENP plus PDT group revealed higher TF expression than NP plus PDT group at post-transcription level (Figure 6B). We also measured ROS levels in BCECs. Dihydroethidium (DHE), one of the most common probes used for superoxide compound anionic fluorescent detection was utilized. As shown in Figure 5, almost no ROS was detected without any treatment (Figure 5A). Red fluorescent signal increased after single radiation was delivered (Figure 5B), but also weak. Significant increase of red fluorescent signal was observed when nanoparticles loaded HMME were added, and remarkablely more ROS was detected in ENP plus PDT group than NP plus PDT group (Figure 5C, 5D). The results demonstrated that PDT induce TF expression of BCECs and conjugation to the surface of nanoparticles significantly increased the TF expression of BCECs post-PDT. Further more, TF expression was consistent with the ROS production, which might suggest more ROS lead to higher TF expression within limits.

### *In vivo* fluorescence imaging of nanoparticles

**Figure 7:**
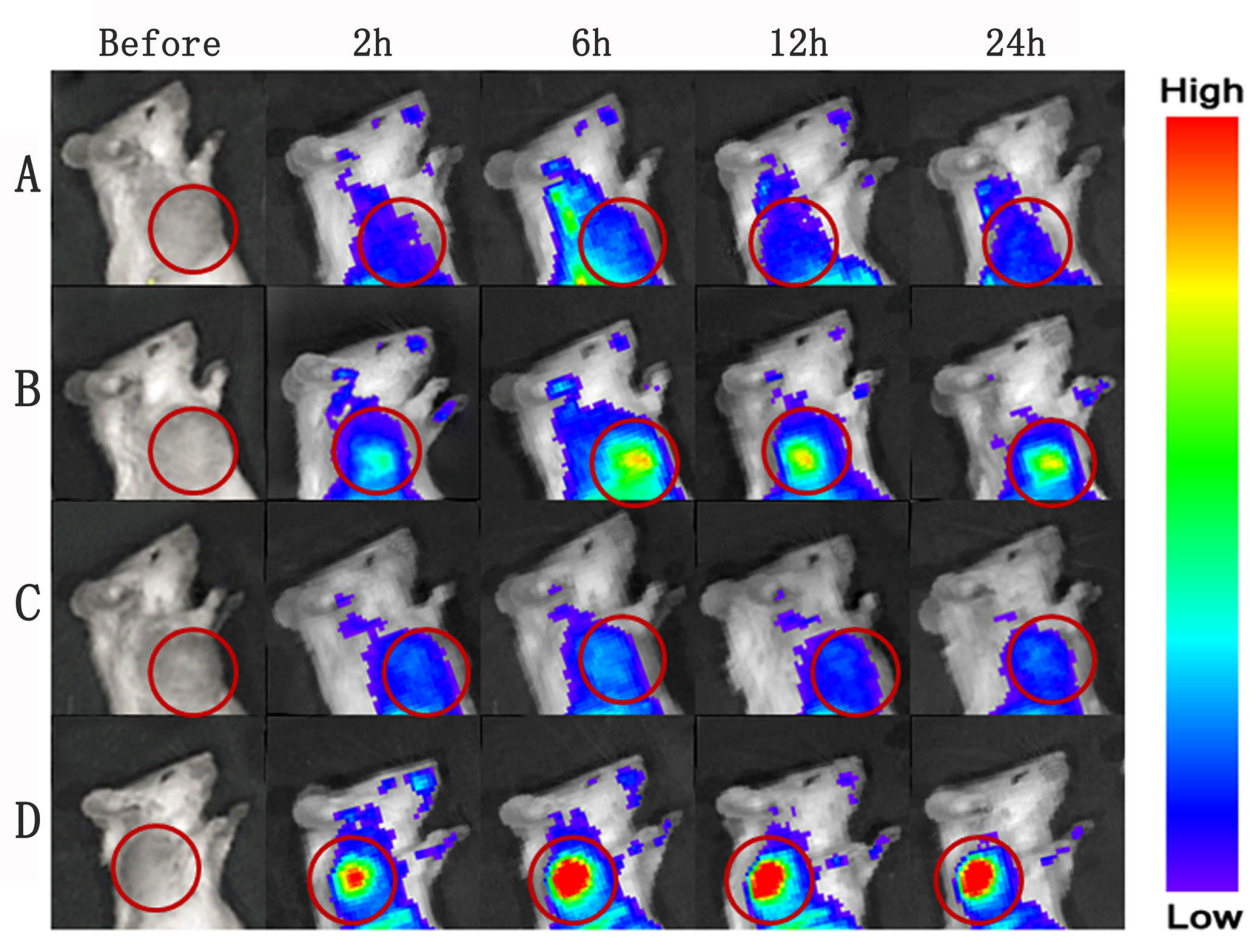
***In vivo*** multispectral fluorescent imaging of tumor-bearing mice at different time points post-PDT. Tumor-bearing mice were respectively conducted with single i.v. administration of nanoparticles **A**., **B**. and a combination of i.v. administration of nanoparticles and PDT **C**., **D**.; Dir-labeled NP was injected *via* tail vein in group A and C, and Dir-labeled EGFP-EGF1-NP was injected in group B and D.

**Figure 8:**
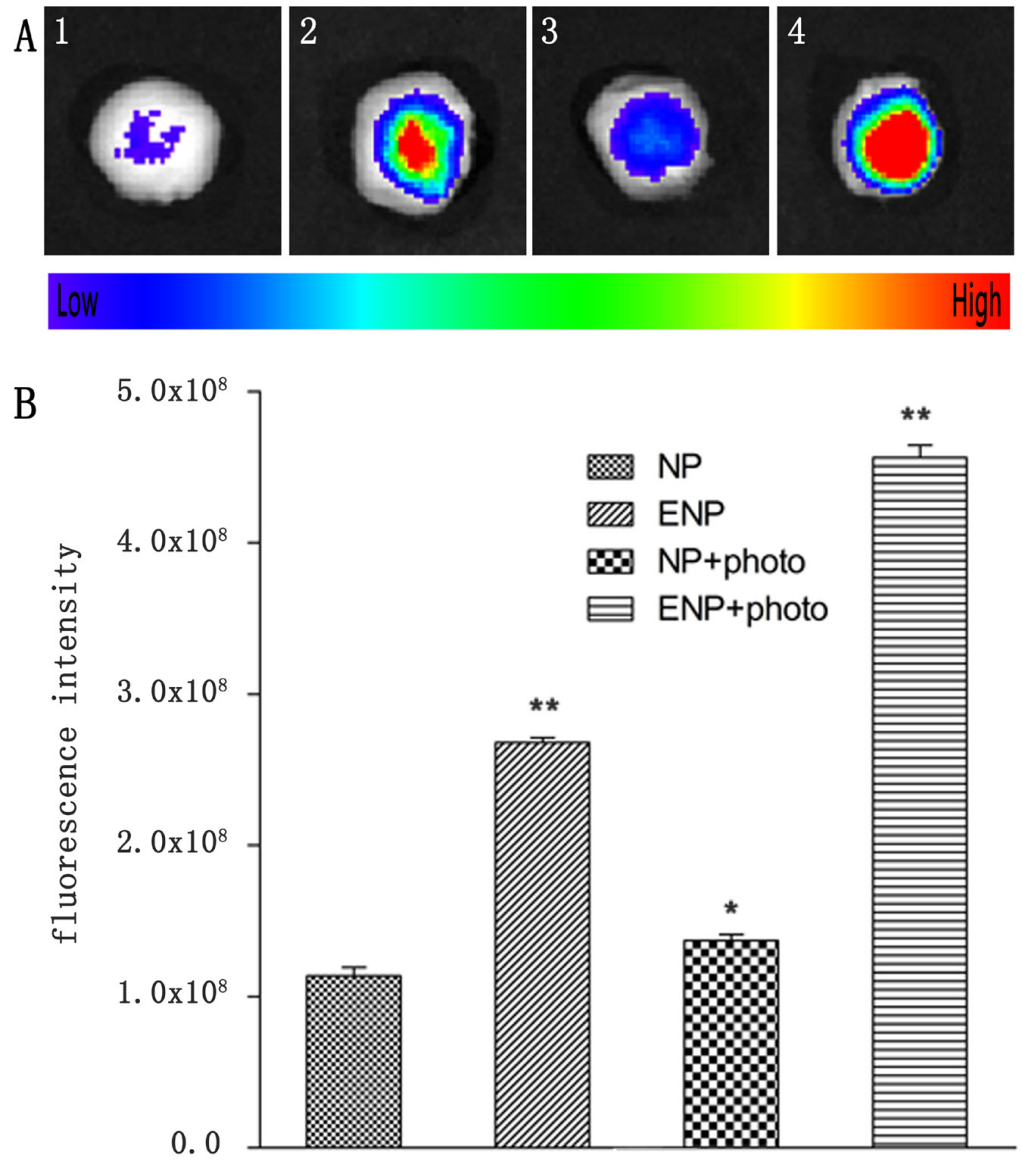
Tumors were incised at 24 h post-PDT for ***ex vivo*** multispectral fluorescent imaging. (1) single i.v. administration of Dir-labeled NP; (2) single administration of Dir-labeled EGFP-EGF1-NP; (3) a combination of i.v. administration of Dir-labeled NP and PDT; (4) a combination of i.v. administration of Dir-labeled EGFP-EGF1-NP and PDT. **B**. corresponding semi-quantitative fluorescence intensities of tumors. Data are expressed as mean ± SEM (*n* = 3); *p < 0.05, **p < 0.01, compared with tomors treated with NP.

The *in-vivo* distribution and tumor accumulation of Dir-labeled-ENP were determined in tumor-bearing mice after PDT using *in vivo* multispectral fluorescent imaging analysis. Dir, a near-infrared dye, was employed as a tag in *in vivo* study, because it tracks nanoparticles in living animals *via* a non-invasive approach. The fluorescence intensity in the tumor of ENP group was significantly higher compared with NP group at any time point ranged from 2 h to 24 h post administration (Figure 7A and 7B). After PDT was delivered, the fluorescence intensity in tumor tissues of the ENP plus PDT group was higher, while the NP plus PDT group showed no significant difference compared to that without PDT groups (Figure 7). The ex-vivo organ imaging also revealed that the accumulation of ENP group in tumor tissues was 2.45-fold higher than that of NP (Figure 8A, 8B). The difference increased to 3.5-fold after PDT was delivered (Figure 8C, 8D). This results further proved that the modification of nanoparticles with EGFP-EGF1 could target TF and therefore improve the nanoparticles in tumor tissues. Further more, PDT further promoted the accumulation of EGFP-EGF1 modified nanoparticles in tumor tissues of tumor- bearing mice.

### Targeted biodistribution of nanoparticles and TF expression of tumor vasculature endothelium in tumor-bearing mice post-PDT in *ex vivo*

**Figure 9:**
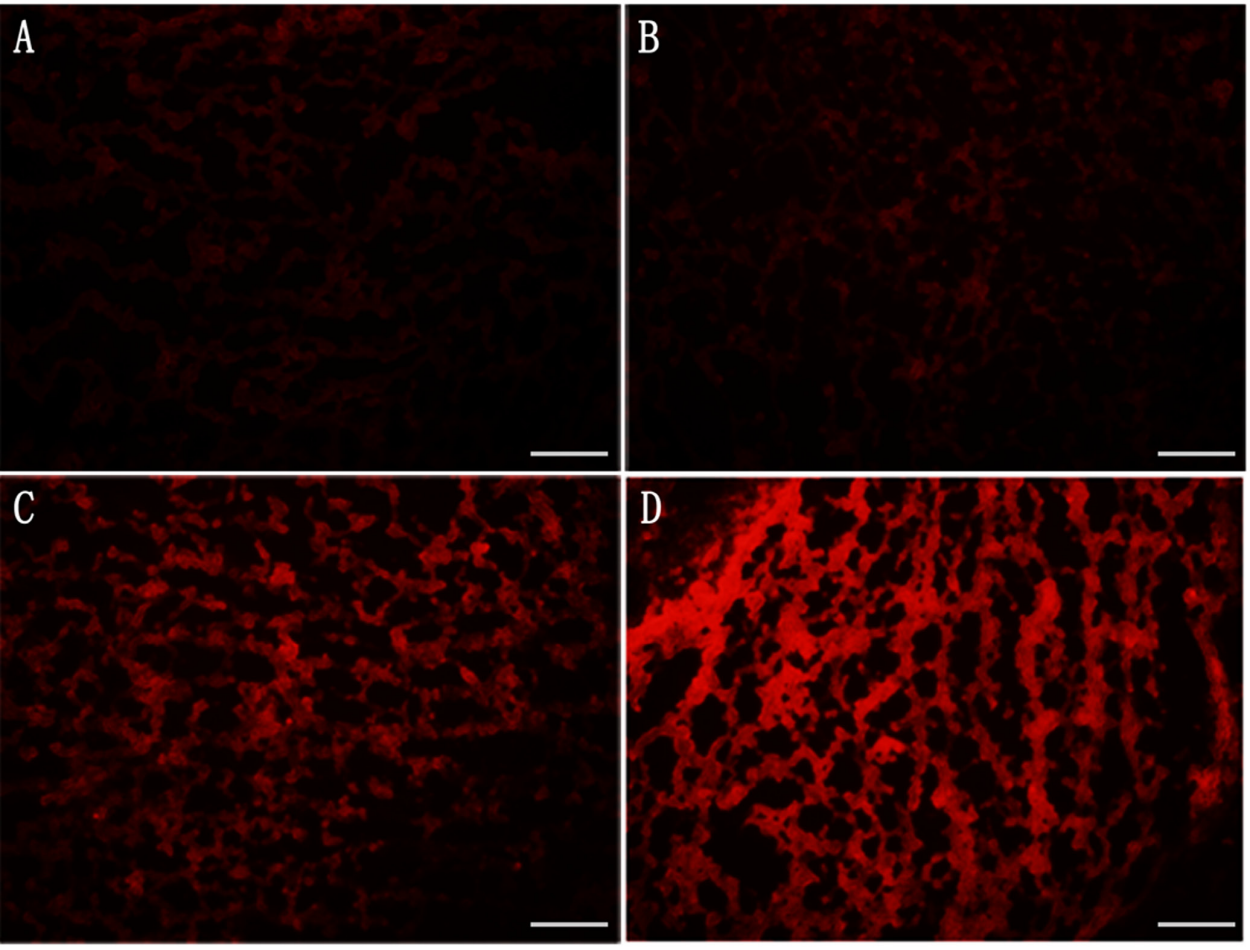
Tumors were harvested for frozen insections at 24h post-PDT and incubated with ROS probe for 30 min at 37 o**C** to estimate ROS production. **A**.control; **B**. single radiation; **C**. a combination of i.v. administration of NP loaded HMME and PDT; **D**. a combination of i.v. administration of EGFP-EGF1-NP loaded HMME and PDT. Bar = 20um.

**Figure 10:**
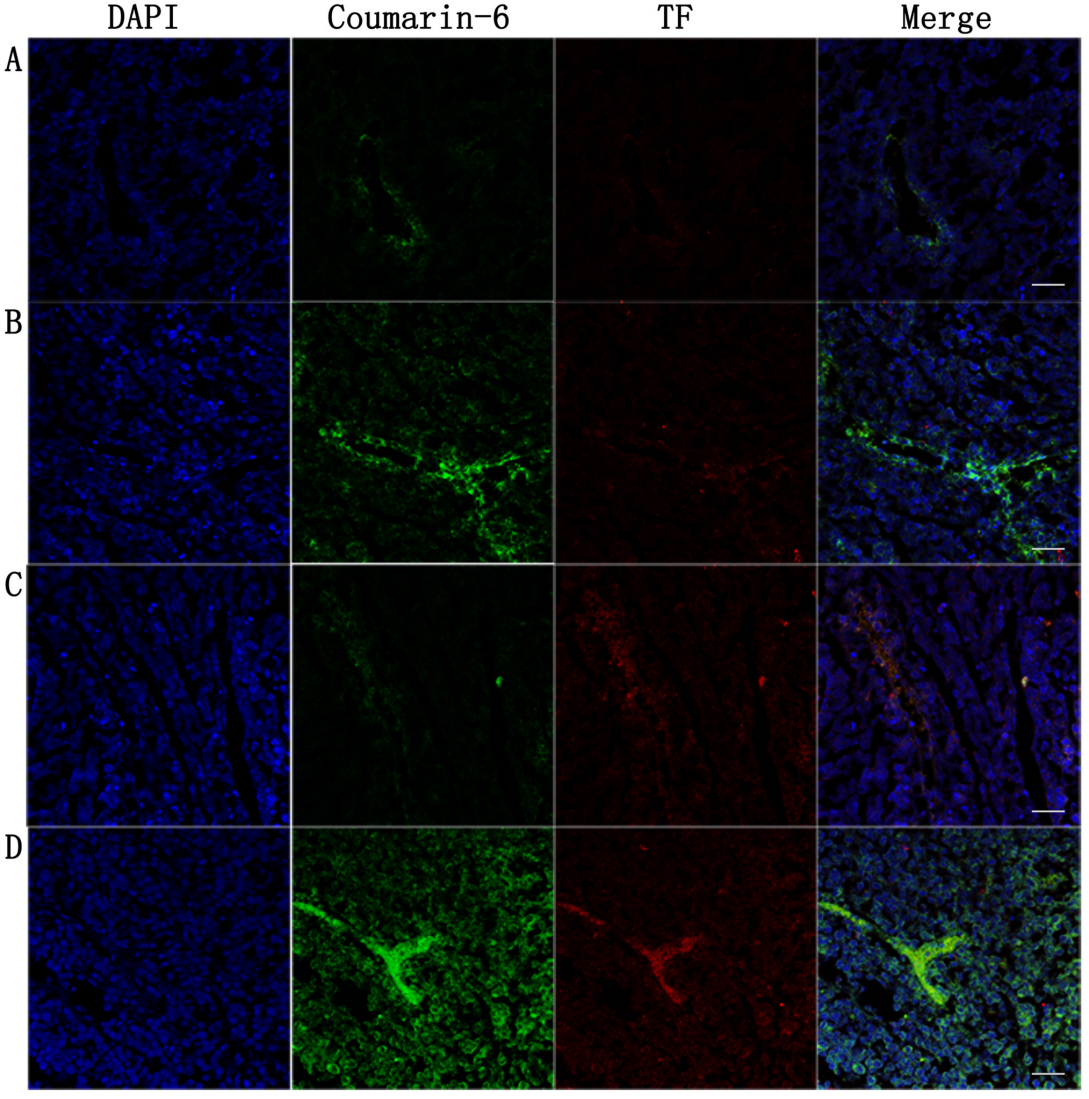
Tumor were harvested for insections at 24h post-PDT to estimate nanoparticles accumulation and TF expression in tumor vessels Tumor-bearing mice were singly administered with nanoparticles**A**.,**B**. or combining i.v. injection of nanoparticles with PDT **C**.,**D**.. Coumarin-6-labeled NP was injected *via* tail vein in group A and C, and coumarin-6-labeled EGFP-EGF1-NP was injected in group B and D. Frozen sections tumors were was stained with rabbit anti rat TF polyclonal antibody examined by confocal microscopy. Blue: cell nuclei. Green: coumarin-6-labeled nanoparticles. Red: TF expression. Bar = 20um.

To investigate the distribution of NPs in tumor *ex vivo*, nanoparticles labeled by coumarin-6 were used as a fluorescent marker at the same time. The tumor were harvested for frozen insections and stained with rabbit anti-rat TF polyclonal antibody and CY3 labeled goat anti-rabbit IgG for observation under confocal microscopy. Frozen insections observation showed the nanoparticles sited in blood vessels more than the other part of local tumor tissues (Figure 10). The biodistribution trend among groups were consistent with *in vivo* fluorescence imaging (Figure 10). TF expressed in the vessels of tumor in both NP group and ENP group, showing equal expression before PDT delivered (Figure 10A, 10B). Increasing TF expression in the vessels of tumor was observed after PDT in both NP plus PDT group and ENP plus PDT group, and the ENP plus PDT group increased more obviously (Figure 10C, 10D). Figure 9 showed that reactive oxygen species detected in tumor tissue were weak and no singnificant difference was observed between control group and single radiation group (Figure A and B). ROS increased prominently after PDT was delivered, and ENP plus PDT group was higher than NP plus PDT group. This results showed that EGFP-EGF1 conjugation contributed to nanoparticles accumulation in tumor vessels. While PDT increased TF expression which might account for ROS.

## DISCUSSION

The vascular endothelium of tumour tissue differs from that of normal tissues in several ways [20], which makes it a potential target for anticancer therapy. Though preexisting abnormal tumor vasculature is more susceptible to the small molecule VDAs according to the differences between normal and tumor endothelium, normal vascular endothelium also seems to be damaged. For example, a cardiovascular toxicity profile was observed in clinical studies [21]. EGFP-EGF1-PEG-PLGA nanoparticles is an established and safe delivery in our previous study. It improves dose-related side-effects [23, 24], meanwhile it provides solutions for solubility problems of poorly soluble drugs and controlled release drug formulations [22]. In this study, EGFP-EGF1 protein was thiolated and conjugated to the malemide covering the PEGylated nanoparticles. Thus formed EGFP-EGF1-NP showed more efficient uptake by abnormal TF expression endothelial cells than NP. This was probably attributed to that EGFP-EGF1 on the surface of nanoparticles which could specifically bind TF. The evidences were as follows. First, BCECs stimulated by TNF-α showed more efficient uptake than unstimulated ones (Figure 3, 4). In view of increasing expression TF of BCECs stimulated by TNF-α (Figure 2), more efficient uptake may because of the combination of EGFP-EGF1 and TF. Second, as shown in Figure 7, ENP group showed remarkably higher accumulation in locally abnormal TF expression tumor than NP group after i.v. administration of nanoparticles. The later sections showed the similar results that tumor vascular endothelium with abnormal TF expression revealed higher accumulation of nanoparticles in ENP group compared with NP group. Furthermore, equal nanoparticles accumulation of tumor vessels was observed between NP group and NP plus PDT group, even though NP group showed higher TF expression of tumor vessels. This result suggest EGFP-EGF1would be a functional protein directing to TF and therefore delivering anti-tumor drugs to tumor vessels. PLGA is one of the most successfully used biodegradable polymers. The US FDA and European Medicine Agency (EMA) have approved it for various drug delivery systems in humans [25]. PEG conjugation improves pharmacokinetics and pharmacodynamics of proteins by increasing protein solubility and stability and also reducing protein immunogenicity and uptake by the reticuloendothelial (RES) system [26, 27]. Since the particle size is an important trait of particles, the size is generally less than 200 nm in diameter for pharmaceutical application. In this study, nanoparticles loaded HMME whether conjugated with EGFP-EGF1 or not were no more than 120 nm (Table 1), which is believed to be favorable to drug carrier. Further, the poor water solubility and low specificity of PSs limited their applications. Therefore various deliveries, such as biodegradable polymeric nanoparticles, ceramic-based (i.e. made of silica) and metallic-based nanoparticles, have been developed to PSs perfect [28, 29]. The surface of the nanoparticles can be functionally attached targeting groups, which will deliver the nanoparticles to cancer cells, to vascular compartments or to cellular sites expressing appropriate receptors. In particular, biodegradable PEG-PLGA nanoparticles have been more fully exploited for the application of drug carrier because they enhance the drug solubility by incorporating water-insoluble PSs into their hydrophobic cores [30].

**Table 1:** The particle size and DLC of NPs and EGFP-EGF1-NPs loaded or non-loaded with HMME, coumarin-6, or Dir

Nanoparticles	Size		DLC(ug/mg)
	Mean size(mean±SD, nM)	PDI	
**HMME-loaded NP**	103.2±2.24	0.166±0.0223	3.75±0.021
**HMME-loaded ENP**	116.4±0.29	0.186±0.0114	4.24±0.016
**Coumarin-6-loaded NP**	106.9±0.14	0.115±0.0093	0.275±0.003
**Coumarin-6-loaded ENP**	117.9±0.22	0.147±0.0143	0.270±0.011
**Dir- loaded NP**	106.6±2.49	0.143±0.0064	1.66±0.005
**Dir- loaded ENP**	118.2±2.20	0.159±0.0037	1.63±0.008

The study for the first time shows a TF-cascade-targeted therapy strategy for anti-vasculature by TF specific PEG-PLGA nanoparticle loaded HMME combining with PDT. PDT is a well-established, clinically approved, minimally invasive approach. It bases on photo-chemistry reaction involving the administration of a photosensitizers (PSs) which produces cytotoxic species under light irradiation and in the presence of oxygen. It has been applied to multiple chorioretinal disorders including age-related macular degeneration, choroidal hemangiomas and central serous chorioretinopathy [31, 32]. It has been approved by FDA for treating cancers like esophageal cancer, non-small cell lung cancer, and skin cancers. It also showed promising results in treating metastasis and local recurrence in clinic trials [33]. Clearance of microorganisms from blood is also investigated and expanded to microorganisms associated diseases, including HPV associated cervical intraepithelial neoplasia [34]. PDT causes tumor ablation *via* direct cytotoxicity, anti-vasculature and activating immune system. Classic PDT depends on direct cytotoxicity and was applied to tumor therapy. Compared with its long DLI, novel anti-vasculature PDT provides relatively short DLI— usually around 15 minutes and has achieved promising results in clinic trials for cancer [35, 36]. Actually, anti-vasculature PDT has been used for AMD clinically and showed promising results. As more tumor endothelial markers have been identified, target PDT was investigated to overcome non-specificity and hydrophobicity of PSs. Current target PDT strategy focused on targeting molecule conjugation with PSs and deliver systems encapsulated PSs. It aimed to target only endothelial cells or target both endothelial cells and tumor cells [7]. In such strategy, PDT is just a trigger for PSs, but in our strategy it's also a trigger for TF cascade. Figure 7 demonstrated that ENP was accumulated obviously higher in tumor after PDT was conducted at time point ranging from 2h to 24h. Figure 8 revealed the same trend quantificationally. Figure 10 showed that after PDT, ENP accumulation in tumor vessels increased significantly. These results suggested that PDT combination with TF specific nanoparticles may have amplified ability to recruit nanoparticles than single TF specific nanoparticles.

It's well known cytotoxic ROS were the predominant effector molecules generated during PDT. And ROS is important in pathway of inducing TF expression. In the study, it's observed that PDT increased TF expression. Figure 10 provided evidence that both NP plus PDT group and ENP plus PDT group showed higher TF expression in tumor vasculature compared with those corresponding groups without PDT delivering. Figure 6 also demonstrated that BCECs expressed higher TF at both transcription and post-transcription level post-PDT. The comprehensive results showed that PDT combination may improve targeting ability of ENP and this enhancing effect may attributed to increasing TF expression of endothelial cells post-PDT. Considering that EGFP-EGF1conjugation contributed to uptake by TF over-expressed endothelium, positive feedback-cascade target strategy was shown——existing available TF to attract ENP-HMME, enhance TF expression *via* PDT damage, then attract more ENP-HMME, thus a positive feedback. What's more, this strategy has precise location efficacy and obviously improves the security. HMME as a new porphyrin-related photosensitizer, has a simpler composition, stronger photoactivity and shorter-term skin toxicity compared with old-generation PSs, such as hematoporphyrin derivatives [37, 38]. They are non-toxic and activated by PDT, and cytotoxic ROS which damage tumor lesions generate. In normal tissue environment, HMME is non-toxic without ROS. However, it release ROS in the diseased area through irradiation activation. Additionally, ROS are restricted to short diffusion distance (10-300 nm according to different estimates) at photosensitizer accumulation site, and have extremely short life time [39, 40]. As imaging technology developes, PDT can highly selectively target the tissues by precise radiation delivery. Two-photon excited PDT has developed to pin-point small volumes at the laser focus, while conventional one-photon PDT excited by visible light can only penetrate relatively superficial lesions. Compounds conjugated porphyrin dimmers based on porphyrin which has been studied for two-photon excited PDT has offer exciting outcomes. Two-photon excited PDT require near-infrared spectrum which is capable of travelling more deeply through lesions than visible light, but also the nonlinear process restricts absorption to the laser focus. Thus greater treatment depths and more highly-precise targeting efficacy may be achieved by two-photon PDT.

So far, many markers have been recognized as targets for tumor vaculature. However, the markers also express on the normal cells. The conventional target therapy which relies on molecule affinity can not achieve truly specific target effect. Non-specific distribution of PSs also limited PDT application. A combination with target delivery with PDT provided an better choice. Moreover, in our strategy, EGFP-EGF1 was used as targeting molecule to TF and PDT triggered TF cascade, which produced a specific tumor blood vessels drug delivery system.

In this study, a TF-cascade-targeted strategy for tumor treatment was developed by combination of TF-targeted HMME-loaded drug delivery system and PDT. It can precisely locate at tumor blood vessels, which depends on TF-targeted HMME-loaded nanoparticles to provide primary localization and then PDT to pinpoint local tumor site to initiate the nanoparticles. *In Vitro* study showed that protein EGFP-EGF1 conjugation to the nanoparticles could significantly contribute to the uptake of nanoparticles by TF over-expressed BCECs as EGFP-EGF1 on the surface of nanoparticles has affinity to TF. As *in vivo* multispectral fluorescent imaging shown, the EGFP-EGF1-NP showed significantly higher accumulation in tumor tissues than NP. Further more, when EGFP-EGF1-NP-HMME was combined with PDT, higher accumulation in tumor blood vessels was observed. The data indicated EGFP-EGF1-NP-HMME has potential to cascade-target tumor vessels by combining with PDT, while have anti tumor vasculature potential.

## MATERIALS AND METHODS

### Materials

The E. coli strain BL21 (DE3) and plasmid pET-28a-EGF1-EGFP were maintained in our laboratory. sodium salt (dye content w90%),DNase I, Coumarin-6 and 40, 6-Diamidino-2-phenylindole (DAPI) were purchased from Sigma Co. (USA). 2-iminothiolane (Traut's reagent), and BCA Protein Assay Reagent were from Thermo fisher scientific inc. (USA). Ni2t-NTA affinity chromatography and Sephacryl S-100 HR chromatography were from GE healthcare (USA). The medium 131/microvascular growth supplement (MVGS), DMEM-F12, collagenase II. The Percoll PLUS was from GE Healthcare Co. (Sweden). Collagenase/dispase and bovine serum albumin (BSA) were purchased from Roche Co. (USA). The Rabbit anti rat TF polyclonal antibody were obtained from Santa Cruz Co. (USA). CY3-labeled goat anti-rabbit IgG were from Abcam Co. (USA). Recombinant rat tumor necrosis factor alpha (TNF-a) was from R&D Systems (USA). Dir (DiIC18 (7) or 1, 1′-dioctadecyl-3, 3, 3′, 3′- tetramethyl indotricarbocyanine Iodide, λex\λem (MeOH) = 748/780 nm) was from Biotium (Hayward, CA,USA). Poly (DL-lacticco-glycolic acid) (50:50) (PLGA, inherent viscosity 0.89, Mww100 kDa) was purchased from Absorbable Polymers (USA). Methoxy-poly (ethylene glycol) (MePEG, MW 3000 Da) was supplied by NOF Corporation (Tokyo, Japan) and Maleimide-PEG (MW 3400 Da) was purchased from Nektar (Huntsville, AL, USA). Hematoporphyrin monomethyl ether (HMME) was ordered from Dibo Chemical Technology Co., Ltd. (Shanghai, China). Sodium cholate was from Shanghai Chemical Reagent Co. (China). Ellman's reagent was from Acros Co. (Bruxelles, Belgium). All the other reagents were commercially available and used without further purification.

### Cell culture and animals

Human Burkitt lymphoma cell lines, CA46 were obtained from American Type Culture Collection and cells were maintained in RPMI-1640 supplemented with 10% fetal bovine serum and 1% penicillin/streptomycin.

Spraguee Dawley rats (50~60g) were provided by the Center of Experimental Animals of Tongji Medical College (Wuhan, China). NOD/SCID mice were provided by HFK Bioscience CO. LTD (Beijing, China). The protocols for treating the animals in the experiment were evaluated and approved by the ethical committee of Tongji Medical College.

### preparation of nanoparticles

PEG-PLGA nanoparticles were prepared *via* an emulsion/solvent evaporation technique and were conjugated with EGFP-EGF1 fusion protein as described previously. HMME-loaded, coumarin-6- or DiR-labelled NPs/ENPs were prepared using the same procedure except that 4mg of HMME, 30 mg of coumarin-6 or 200 mg of DiR was additionally added to dichloromethane containing copolymers before primary emulsification.

### Characterization of nanoparticles

The morphology of the nanoparticles were examined by transmission electron microscopy (TEM, Hitachi, Japan) after negative staining with 1% sodium phosphotungstate solution. A Zeta Potential/Particle Sizer NICOMP™380 ZLS (Pss. nicomp particle size system, USA) measured the mean diameter of the nanoparticles by dynamic light scattering (DLS) with He-Ne laser at 632.8 nm. Concentrations of HMME and coumarin-6 were determined based on their absorbance spectra in acetonitrile using established standard curve line. The Dir concentrations were measured by UV spectrophotometry at a wavelength of 700 nm. Drug loading capacity (DLC) was determined based on the ratio of final drug weight to overall weight of the nanoparticles.

### *In vitro* uptake of nanoparticles

BCECs were obtained from the brain of SD rat *via* being minced into small pieces and then digested by collagenase, following by gradiently centrifuging using percoll, according to previously described techniques. When it came to the 3rd passage, BCECs were seeded onto sterile coverslips in 6-well plates and incubated with tumor necrosis factor-α (TNF-α) of 100ng/mL, after which the western blotting technique and real-time PCR were conducted to examine TF expression as previously described.

BCECs were co-incubated with TNF-α and coumarin-6 labeled NP-HMME,ENP-HMME (50ng/ml of coumarin-6) relatively for 3 hours for the examination of intracellular uptake. Then cells were washed. After that, BCECs were observed under fluorescence microscope for qualitative analysis. For quantitative analysis of uptake efficiency,BCECs were stained with DAPI and digested for flowcytometry.

### *In-vitro* quantification of TF expression of BCECs post-PDT

1×10^6^ BCECs grown overnight were incubated with NP-HMME and ENP-HMME (50ng/ml) for 3 hours. Before PDT, nanoparticles-containing medium was replaced with fresh medium. Then PDT was conducted on BCECs by irradiating with cold light source. Cells were then washed and incubated with DHE for 30min at 37 °C for observing intracellular ROS under fluorescence microscope.

To investigate the expression level of TF by BCECs post-PDT, cells were incubated with NP-HMME and ENP-HMME (50ng/ml) for 3 hours and then PDT was conducted. At 2 hours post-PDT, cells were washed, lysed with RIPA and assessed for TF expression by western blotting and real-time PCR.

### Mouse model and *In vivo* PDT

Mice were inoculated with intradermal injection of 1×10^7^ CA46 cells over the depilated right shoulder of Nod/SCID mice (4-5 weeks old). PDT was conducted when tumors reached a diameter of 5 to 7 mm (28 days after inoculation).

Tweenty mice were divided into five groups at random. Phosphate buffer saline (2 ml/kg), ENP-HMME administration (dose 4 mg/kg, reconstitution in phosphate buffer saline), NP-HMME administration (dose 4 mg/kg, reconstitution in phosphate buffer saline), ENP-HMME administration (dose 4 mg/kg, reconstitution in phosphate buffer saline) plus PDT, NP-HMME administration (dose 4 mg/kg, reconstitution in phosphate buffer saline) plus PDT. Phosphate buffer saline and nanoparticles were injected into the tail veins right after model accomplished. PDT delivery was 10 minutes after intravenous injection. The fiber optic bundle of a cold light source (KL 1500 LCD, SCHOTT, Germany) with a 4.5 mm aperture was positioned directly above tumors of mice lying on the left side. PDT was delivered for constant 15 minutes when mice were anesthetized by intraperitoneal injection of 5% chloral hydrate.

### Fluorescence imaging of nanoparticles post-PDT in tumor-bearing mice

Nanoparticles loaded HMME were labeled by Dir (dose 0.8 mg/kg, reconstitution in physphate buffer saline) for investigation of pharmacokinetics study in tumor-bearing mice. At various points (2h, 6h, 12h, 24h) post-PDT, fluorescent scans were conducted on anesthetized mice using Kodak *In-vivo* Multispectral Imaging system (Carestream Health, USA). Mice were euthanized and main organs were harvested at 24h post-PDT. Each organ was washed with PBS and then Maestro 2 *In Vivo* Imaging System (CRI, USA) was used for capturing fluorescence images. The wavelength was 780 nm with a deep red filter.

### Biodistribution of nanoparticles and TF expression of tumor vasculature endothelium in tumor-bearing mice post-PDT

Nanoparticles loaded HMME were labeled by coumarin-6 (dose 80 ug/kg, reconstitution in phosphate buffer saline) for observing nanoparticles distribution and TF expression. Animals were killed at 24h post-PDT. Tumors were collected for frozen sections of 5 um thickness and stained with rabbit polyclonal to tissue factor. And then sections were observed under confocal microscopy to investigate nanoparticles distribution and TF expression of blood vessel. Tumors were excised right to analyze ROS by incubation with probe DHE for 30 minutes at 37°C. Fluorescence slides were observed under a confocal microscope.

## Abbreviations

PSs: photosentitizers
PDT: photodynamic therapy
TF: tissue factor
ROS: reactive oxygen species
NP: PEG-PLGA nanoparticle
ENP: EGFP-EGF1-NP
BCEC: brain capillary endothelium cell
HMME: Hematoporphyrin monomethyl ether
