# Correction: A tissue factor-cascade-targeted strategy to tumor vasculature: a combination of EGFP-EGF1 conjugation nanoparticles with photodynamic therapy

**DOI:** 10.18632/oncotarget.25079

**Published:** 2018-04-03

**Authors:** Wei Shi, Yanxue Yin, Yao Wang, Bo Zhang, Pei Tan, Ting Jiang, Heng Mei, Jun Deng, Huafang Wang, Tao Guo, Zhiqing Pang, Yu Hu

**Affiliations:** ^1^ Institute of Hematology, Union Hospital, Tongji Medical College, Huazhong University of Science & Technology, Wuhan 430022, China; ^2^ Targeted Biotherapy Key Laboratory of Ministry of Education, Wuhan, Hubei, China; ^3^ Department of Pediatrics, Tongji Hospital, Tongji Medical College, Huazhong University of Science & Technology, Wuhan 430022, China; ^4^ Department of Pharmaceutics, School of Pharmacy, Fudan University, Shanghai, China

**This article has been corrected:** The proper name of the institution is as follows:

^**1**^ Institute of Hematology, Union Hospital, Tongji Medical College, Huazhong University of Science & Technology, Wuhan 430022, China

Original article: Oncotarget. 2017; 8:32212-32227. https://doi.org/10.18632/oncotarget.12922

